# Supplementary material for: Asynchronous magnetic resonance elastography: Shear wave speed reconstruction using noise correlation of incoherent waves
Source: Magn Reson Med. 2022 Oct 27;89(3):990–1001. doi: 10.1002/mrm.29502 (PMC9792433; doi:10.1002/mrm.29502)
Supplement: Supplementary file 1 — DATA S1 MATLAB code used to generate the numerical simulations of Figure 1 [file MRM-89-990-s002.zip › k-Wave/helpfiles/example_pr_3D_tr_planar_sensor.html]

3D Time Reversal Reconstruction For A Planar Sensor Example (k-Wave)


# 3D Time Reversal Reconstruction For A Planar Sensor Example

This example demonstrates the use of k-Wave for the reconstruction of a three-dimensional photoacoustic wave-field recorded over a planar array of sensor elements. The sensor data is simulated and then time-reversed using `kspaceFirstOrder3D`. It builds on the 3D FFT Reconstruction For A Planar Sensor and 2D Time Reversal Reconstruction For A Line Sensor examples.

- Open the file in the MATLAB Editor
- Run the file in MATLAB

## Contents

- Performing the reconstruction

## Performing the reconstruction

The sensor data is simulated using `kspaceFirstOrder3D` in the same way as in preceding examples. The utilised initial pressure distribution and the sensor mask are shown in 3D FFT Reconstruction For A Planar Sensor. The time-reversal reconstruction is then performed by assigning the time varying pressure recorded over the detector array to `sensor.time_reversal_boundary_data`.

```
% run the simulation
sensor_data = kspaceFirstOrder3D(kgrid, medium, source, sensor, input_args{:});

% reset the initial pressure
source.p0 = 0;

% assign the time reversal data
sensor.time_reversal_boundary_data = sensor_data;

% run the time-reversal reconstruction
p0_recon = kspaceFirstOrder3D(kgrid, medium, source, sensor, input_args{:});
```

A plot of the reconstructed initial pressure distribution with a positivity condition is shown below.
